# Ocular and corneal aberrations changes in controlled randomized clinical trial MiSight® Assessment Study Spain (MASS)

**DOI:** 10.1186/s12886-021-01865-y

**Published:** 2021-03-01

**Authors:** Daniela Lopes-Ferreira, Alicia Ruiz-Pomeda, Belén Peréz-Sanchéz, António Queirós, César Villa-Collar

**Affiliations:** 1 CEORLab, Centre of Physics, Clinical and Experimental Optometry Research Lab, University of Minho, Campus de Gualtar, 4710-057 Braga, Portugal; 2 Servicio de Oftalmología. Hospital Universitario de Mostoles, Mostoles, Madrid, Spain; 3 Department of Statistics, Mathematics and Informatics, Area of Languages and Computer Systems, Miguel Hernández University of Elche, Elche, Alicante, Spain; 4 Department of Pharmacy, Biotechnology, Optics and Optometry, European University of Madrid, C/Tajo s/n, 28670 Villaviciosa de Odón, Madrid, Spain

**Keywords:** MiSight, Myopia, Contact lenses, Axial growth, Children

## Abstract

**Background:**

To compare ocular and corneal inherent aberrations in the naked eyes of randomly selected children fitted with MiSight contact lenses (CL) for myopia control, versus children corrected with single-vision spectacles (control), over a 24-months period.

**Methods:**

Children aged 8 to 12 years, with myopia (-0.75 to -4.00 D sphere) and astigmatism (< -1.00 D cylinder) were randomly assigned to the lens study group (MiSight) or the control group (single-vision spectacles). The root mean square aberration (RMS) was determined as corneal (RMS_C), corneal high order RMS (HO_RMS_C), corneal low order RMS (LO_RMS_C), ocular (total) RMS (RMS_T), ocular high order RMS (HO_RMS_T), ocular low order RMS (LO_RMS_T), corneal spherical aberration (SA_C) and ocular SA (SA_T) were calculated by aberrometry measures at the baseline, on 12-months and 24-months visits. A 5 mm diameter was defined for the analysis in all visits for all subjects. Only the dominant eye was analyzed.

**Results:**

Seventy-four subjects completed the clinical trial: 41 subjects from the MiSight group (age: 11.01 ± 1.23 years) and 33 from the single-vision group (age: 10.12 ± 1.38 years). RMS_T significantly changed (0.57 ± 0.20 µm, *p* = 0.029) after 24-months in the control group. In the MiSight group no significant changes were registered (*p* > 0.05). The SA_C and SA_T did not reveal significant changes between visits or between groups (*p* > 0.05).

**Conclusions:**

Along 2 years, MiSight CL did not induce significant changes in RMS of anterior cornea or total ocular RMS. Contrary, in control group the RMS_T significantly changed as response of greater eye growth and myopia progression. The results obtained in present study allow to predict corneal or total aberration changes, in children, in response of wearing of MiSight lens along the time.

**Trial registration:**

: ClinicalTrials.gov Identifier: NCT01917110.

## Background

Axial elongation promotes myopia, causing longer eyes than in the non-myopic state. The exacerbated axial elongation can lead to pathological issues such as early cataract,[1] glaucoma[2] and retinal detachment[3] which may eventually end in permanent and irreversible visual impairment.[4] Axial length (AL) increases in myopic eyes producing axial negative refractive error and hyperopic relative peripheral refractive error.[5] Myopic patients usually present greater accommodative lag (or central hyperopic defocus) during close-vision tasks compared with hyperopic eyes. The wearing of progressive lenses in children has been shown to produce a reduction in accommodative demand and accommodative lag, i.e. the central hyperopic defocus, thus preventing eye elongation and myopia progression in certain cases (around 15 %).[6–8] Animal studies, using monkeys for example, have shown that hyperopic retinal defocus[9] and peripheral hyperopic defocus alone[10] can produce a defocused image in different regions of the retina, stimulating the axial growth of the eye in an attempt to achieve a clearly focused image on the retina.

Through magnetic resonance imaging, it has been possible to characterize the myopic retinal posterior shape as less oblate than the hyperopic retinal shape.[11] A population with a high prevalence of ametropia (*n* = 313) was studied to understand the interaction between higher order aberrations (HOAs) and refractive error, and the results showed less positive values (median 0.07µm, ranged 0 to 0.11µm) of 4th order spherical aberrations (SA) in longer/myopic eyes ( median of M=-0.75D and M=-1.13D) in patients with age vary between 9 and 10 years and age 15–16 years.[12] Another recent study has also revealed that less AL growth was observed in subjects with more positive SA.[13].

In the last decade, the hypothesis that peripheral hyperopia causes myopia has been well investigated. Accordingly, several studies tested optical interventions to reduce the central hyperopic defocus (accommodative lag) and peripheral hyperopic defocus, essentially by wearing center-distance multifocal contact lenses (MFCL)[14, 15] or by orthokeratology.[16–18] However, despite the reduction of peripheral hyperopia effectively preventing ocular growth, with an efficacy varying between 30 %[19] and 56 %,[17] it is not clear if myopia is only regulated by the relative peripheral defocus. As seen above, the HOA, namely SA, could also eventually be involved in the process of controlling ocular growth in myopia.

In 2018, around 0.7 % of the contact lenses prescribed worldwide were soft contact lenses for myopia control.[20, 21] Different designs of MFCL achieved different rates in controlling eye elongation and different changes on ocular aberrations induced depending of the design.[22–24] In terms of best corrected visual acuity (BCVA), MiSight has recently been shown to provide satisfactory visual acuity at long and short distance.[25].

Based on the above-mentioned facts, the present study consists of comparing the aberrations in the eyes of children randomly allocated to a control group using single-vision (SV) spectacles, and a study group in which children wore a myopia control MiSight lens over a period of 24-months. The main objective of the study was to determine the differences between the ocular and corneal aberrations arising from the myopia progression in both control and study groups.

## Methods

This study is part of the MiSight® Assessment Study Spain (MASS),[26–29] designed to assess the efficacy and subjective acceptance of MiSight® CL versus distance SV spectacles in myopic children over a 24-months period. The protocol was approved by the CEI-R (Regional Research Ethics Committee of the Community of Madrid, Spain) and adheres to the tenets of the Declaration of Helsinki. The clinical trial was registered in Clinical Trials (ClinicalTrials.gov Identifier: NCT01917110), where the outcome measures and eligibility criteria may be consulted. After receiving an explanation of the nature and possible consequences of the study, all parents provided signed permission for their children to take part, and the participants themselves also provided written consent.

Healthy subjects of European descent, being 8 to 12 years of age, with moderate levels of myopia (-0.75 to -4.00 D) and astigmatism (< -1.00 D), and free of systemic or ocular disease, were recruited for this study. On the baseline initial visit, all subjects underwent a full anterior segment examination, indirect fundus microscopy, binocular-vision and refractive evaluation. Eligible subjects were sequentially randomized into either the study group (MiSight CL) or the control group (single-vision spectacles, Shamir, Spain).

Subjects included in the CL group were instructed to wear their lenses for at least 6 days per week, but not exceeding 15 hours per day. The CL group returned for follow-up assessments after 1 week, and then at 1, 6, 12, 18 and 24–months intervals. Subjects in the control group were prescribed standard, single-vision, spherocylindrical spectacles (Monofocal Shamir Alite, 1.56 HMC) and were asked to wear the spectacles at all times. The spectacle group was asked to return for follow-up assessments at 6, 12, 18 and 24-months intervals.

Seventy-nine subjects were recruited for the study between September 2013 and June 2016. Forty-six children (58 %) were randomly allocated to the MiSight group (study group) and 33 to the single-vision spectacles group (control group). A total of five individuals dropped out during the 24-month period of the study and the reasons for this have been discussed in a previous study. [27].

### MiSight contact lenses

MiSight is a soft (hydrophilic) CL daily disposable contact lens composed of Omafilcon A material. Remaining technical characteristics and a more exhaustive explanation of its characteristics can be found in our last study.[27] MiSight was recently approved as the first soft contact lens indicated to slow the progression of myopia in children by the FDA.[30].

### Wavefront evaluation

As in baseline, during 12-months and 24-months follow-up visits, the measurements were obtained using the I-Profiler plus (Carl Zeiss, Germany) that is a wavefront sensor using the Hartman-Shack principle. The subjects of the control group (SV) and the study group (MiSight CL) were assessed without any optical compensation, in a dark room to guarantee wide pupil sizes. Correspondingly, the subjects from the study group were instructed to remove their lenses 30 minutes before the evaluation, and the subjects from the control group removed their spectacles.

The subjects were instructed to fix the apparatus stimulus (the image of a balloon), while the infrared light beam generated a point of light on the subject´s retina, and then the light crossed the optic system of the subject’s eye between the retina and corneal surface, thus generating a wavefront captured by a Hartmann-Shack sensor. The software algorithms proceeded to calculate a two-dimensional and chromatically-coded map of the wavefront by a comparison between the points of light generated by array of microlens and a reference point. The quadratic value of all aberrations (RMS) was registered, as well as the average of the quadratic value of high order (HO_RMS) and low order (LO_RMS) aberrations at ocular and corneal level, in microns (µm). Spherical aberrations of the eye and cornea were also obtained. The corneal aberration analysis was obtained by derivation of corneal topography data given directly from apparatus, being the aberrometric profile of cornea correspondent to the anterior surface of cornea. In this work, the corneal aberrations refer to only anterior corneal aberrations. Only data from the dominant eyes were analyzed and the ocular dominance was determined by imposition of + 1.50D lens method. All data have been rescaled to a 5 mm pupil size for comparison purposes.[31].

### Statistics

Statistical analysis of data was performed using the SPSS statistical software package SPSS 18 for Windows. For the analysis, data for children who attended the 24-months visit were included in the analysis. The level of statistical significance was taken as 5 % (significance *p* < 0.05).

The differences between the two study groups were analyzed for baseline biometric and aberration data, using the analysis of variance (ANOVA) and the test F Brown-Forsythe, depending on the result of the contrast homogeneity of variance performed with Levene’s test. In addition, the Kruskal-Wallis test was performed if the results of Kolmogorov-Smirnov had led to the rejection of the hypothesis of normality of the data for pairwise comparisons.

## Results

Table 1 presents the initial biometric and aberration data. Both groups studied showed similar initial axial length (*p* = 0.603), basal corneal and total RMS (*p* > 0.05 for both parameters). None of the remaining parameters represented in Table 1 showed significant differences (*p* > 0.05).

**Table 1:** Comparison of baseline parameters of biometry and optical quality expressed as mean ± standard deviation (SD) for subjects who completed the study up to 24-months

	Control group (*n* = 33)	MiSight group (*n* = 41)	*p*-value
Natural PUP Ø (mm)	5.58 ± 0.69	5.74 ± 0.71	0.307
RMS_T (µm)	2.40 ± 1.02	2.57 ± 1.01	0.484
LO_RMS_T(µm)	2.38 ± 1.02	2.56 ± 1.01	0.479
HO_RMS_T (µm)	0.20 ± 0.08	0.22 ± 0.10	0.314
RMS_C (µm)	0.65 ± 0.38	0.70 ± 0.35	0.529
LO_RMS_C (µm)	0.51 ± 0.29	0.62 ± 0.32	0.182
HO_RMS_C (µm)	0.30 ± 0.31	0.28 ± 0.25	0.794
SA_T (µm)	0.03 ± 0.06	0.06 ± 0.07	0.110
SA_C (µm)	0.13 ± 0.11	0.12 ± 0.04	0.732

*T* total; *C* corneal; *LO* low order; *HO* high order; *SA* spherical aberration; *PUP Ø* pupil diameter measured by pupilometer

Aberration values calculated to constant 5 mm pupil diameter analysis

Control group (spectacles); MiSight group (MiSight CL)

*p*-value: refers to the statistical *p*-value. T-test comparison between means

After 24-months in the control group, RMS_T and LO_RMS_T increased (+ 0.57 ± 0.20 µm and + 0.46 ± 0.18 µm) RMS_T significantly (*p* = 0.029) and LO_RMS_T slightly (*p* = 0.047) (Table 2). A consistent increase in values of RMS_T and LO_RMS_T was verified in both groups, although only in the control group did there occur significant differences between 12-months and 24-months, and between the baseline and 24-months (*p* < 0.05).

**Table 2:** Changes (mean ± SD) in corneal (C) and total (T) RMS to low order (LO) and high order (HO) and spherical aberration (SA) at each visit of subjects who completed the study up to 24-months

	Control group (*n* = 33)				MiSight group (*n* = 41)				Difference among groups along 24-months period (mean) Control-MiSight
	Baseline	12 months	24 months	*p*–value	Baseline	12 months	24 months	*p*–value	
RMS_T (µm)	2.40 ± 1.02	2.70 ± 0.98	2.95 ± 1.03	**0.029**	2.57 ± 1.01	2.70 ± 0.96	2.96 ± 1.13	0.081	0.29
LO_RMS_T (µm)	2.38 ± 1.02	2.69 ± 0.99	2.91 ± 1.03	**0.047**	2.56 ± 1.01	2.68 ± 0.97	2.95 ± 1.13	0.091	0.19
HO_RMS_T (µm)	0.20 ± 0.08	0.23 ± 0.16	0.19 ± 0.06	1.000	0.22 ± 0.10	0.20 ± 0.09	0.20 ± 0.08	1.000	0.02
RMS_C (µm)	0.65 ± 0.38	0.60 ± 0.23	0.62 ± 0.28	1.000	0.70 ± 0.35	0.76 ± 0.26	0.79 ± 0.33	0.620	-0.10
LO_RMS_C (µm)	0.51 ± 0.29	0.54 ± 0.24	0.54 ± 0.26	0.920	0.613 ± 0.38	0.70 ± 0.26	0.74 ± 0.28	0.055	-0.07
HO_RMS_C (µm)	0.30 ± 0.31	0.23 ± 0.09	0.26 ± 0.17	1.000	0.28 ± 0.25	0.27 ± 0.14	0.27 ± 0.20	1.000	-0.02
SA_T (µm)	0.03 ± 0.06	0.05 ± 0.06	0.04 ± 0.06	0.430	0.06 ± 0.07	0.06 ± 0.06	0.06 ± 0.09	1.000	0.01
SA_C (µm)	0.13 ± 0.11	0.11 ± 0.03	0.10 ± 0.05	1.000	0.12 ± 0.04	0.13 ± 0.05	0.11 ± 0.05	1.000	-0.02

*T* total; *C* corneal; *LO* low order; *HO* high order; *SA* spherical aberration;

Aberration values calculated to constant 5 mm pupil diameter analysis

Control group (spectacles); MiSight group (MiSight CL)

*p*-value: refers to the statistical *p*-value. Bonferroni Test to comparison baseline vs. 24 months

Bold p-values are statistically significant (p < 0.05)

The baseline values of SA_T and SA_C were positive in both groups, being 0.03 ± 0.06 µm and 0.13 ± 0.11 µm respectively in the control group, and 0.06 ± 0.07 µm and 0.12 ± 0.04 µm in the MiSight group. On the 12- and 24-months follow-up visits, the values of SA_T and SA_C did not change significantly in either of the groups (*p* > 0.05), compared with the baseline.

## Discussion

The current study shows the evolutions of aberrations in the eye and in the anterior cornea of children corrected with dual-focus CL and single-vision spectacles. The measures were obtained after take-off the optical compensation (naked eye), reversely to other studies that have evaluated the aberrations or the light disturbance with CL designed for myopia control in the eye.[22, 32] The novelty of this work is centered in study of structures/ tissues dynamics in adaptation or response to different optical compensations.

Several authors such as Fedtke et al.[33] studied center-distance design MFCL, which previously were considered favorable to myopia control by producing peripheral relative myopia showing that also significantly induce positive shifts in primary SA (4th order SA). The absolute value of SA increased according to the increase of pupil size and add power. Dual-focus designed lenses also produced positive SA in 3 mm pupil size, but conversely, in greater pupil sizes of 4 mm, a higher negative value of 4th order SA was found, and even more so in the 5 mm pupil size. These findings show that the changes in HOA induced by dual-focus designs (such as MiSight) are substantially more pupil-dependent than the center-distance MFCL designs[31, 34] and shows the role of SA whose magnitude differently affects the myopia control.[34].

Previous MASS studies have also reported an increase on monocular light disturbance perception[28] and near-vision worsening[26] in subjects wearing MiSight compared with subjects wearing single-vision spectacles (control group). Nevertheless, over 2 years of follow-up there could be seen a significant binocular attenuation effect, and a consequent decrease in the monocular light disturbance. Fernandes et al.[35] did not find significant differences between manifest light disturbance in presbyopes after 15 days in participants who had been wearing Biofinity MF compared with monovision CL. In addition, there were no significant changes in the Quality of Vision questionnaire between different CL modality, suggesting an objective and subjective adaptation to multifocality over time. Another previous study also found satisfactory high-contrast distance and near visual acuity (VA) (LogMAR about 0.00 or better) with both lens modalities (monovision and MFCL) being highly comparable with the best corrected VA in spectacles at both long and short distance.[36].

In the present study, contrary to other studies, we performed measurements on the condition of the naked eye. For this reason, we were able to perceive the changes at the HOA level promoted by changes occurring in the eye, essentially through the influence of CL in the cornea and by the axial elongation during the myopia progression (with and without influence), this could be pointed as a limitation. But are known the difficulties in measuring total aberrations in multiconcentric multifocal CL on eye as aliasing effect or errors associated with measuring areas of power transition.

The data of present study shows that after 2 years only the RMS_T significantly increased in the control group, suggesting that the RMS_T increases more in the control group promoted by greater increase of AL and myopia.[27].

There has been several previous studies regarding the relation between HOA and refractive error. In a recent review, Hughes et al.[34] considered this topic controversial and Little et al.[12] explained this relation due mainly asymmetries in sample size, differences in subjects’ age/ethnicity, or varying methodological procedures, including differences in classification of refractive error. Despite this, most studies[37, 38] show that HOA values are higher in myopes than emmetropes or hyperopes, and there is a correlation between primary SA (4th order) and refractive error in such a way that value of 4th order SA in the eye becomes less positive with increasing myopia and axial length.[13] The present study did not reveal significantly changes in SA of the eye during the 2-year period of evaluation, during which myopia progressed asymmetrically in both groups. Despite differences in terms of axial elongation between the groups studied, none showed tendency to SA values becoming less negative over the 2 years. These findings indicate that the SA of the eye (SA_T) firstly evolved differently with AL growth in our sample compared with other larger Asiatic samples[13], and secondly the greater fluctuation occurred in the control group throughout the evaluation, although differences were no significant.

The pupil diameter is an important factor in multifocal CL performance, because the variation of light makes to vary the power areas activated by light. By this reason it is expected that depending the illumination in the environment, children will be subject to different level of aberration. As several previous studies use 5mm pupil diameter, we chosen it.[12, 22].

The baseline myopia of the subjects allocated to the MiSight group was slightly higher (*p* > 0.05) than in the control (− 2.16 ± 0.94 vs. − 1.75 ± 0.94 D) as these subjects were older (*p* < 0.05); for this reason, in the MiSight group there were slightly longer eyes (24.09 ± 0.55 vs. 24.00 ± 0.86 mm),[27] in present study we obtained higher RMS_T (2.57 ± 1.01 vs. 2.40 ± 1.02 µm) and more positive SA_T (0.06 ± 0.07 vs. 0.03 ± 0.06 µm) in control group (*p* > 0.05 to all). Considering the findings from Little et al.[12], this group, by having longer eyes, should present less positive values for primary SA, which was not verified in this study. With regard to axial length, however, at the end of 2 years, differences between the groups had already become significant (*p* < 0.05), the axial length in the control group being 0.16 mm longer and the value of SA less positive. In other studies, a tendency was seen among increase of the axial length/myopia shift and the basal SA of the eye[13, 39]; our results, reversely showed that it is not clear the correlation between high positive SA values and low myopic shift occurred in the MiSight group.

In the current study, the axial elongation retention rate at 2 years was 36 %[27] lower than that found in another recent study with a similar methodology (53 %).[40] However, this asymmetry can be explained in relation to differences in age/AL among the groups at baseline as explained above.

The center-distance MFCL and orthokeratology,[41] by reducing the peripheral hyperopia, produce positive SA[42] that is known to be effective in myopia retention, and is inducted by contact lenses.[43] The present study looked at different changes in aberrations in myopic children treated with dual-focus MFCL vs. spectacles. If, on the one hand, it is known that different commercially available designs of MFCL worn on the eye differently affect the magnitude of the peripheral refraction and SA profiles along different visual field meridians,[44] on the other hand, it is certain that its effectiveness depends on multiple factors as the inherent aberration/SA, the age and the pupil size present in the eye at the beginning of treatment.

At corneal level, no significant changes were seen suggesting that the wearing of MiSight in children’s eyes did not produce any influence on corneal shape, resulting in no changes in corneal aberrations, even after 24-months of daily use of approximately 11 hours per day, 6 days per week.[27] The safety and acceptance of MiSight has already been evaluated[45] over 3 years and does not report significant adverse events in children between 8 and 12 years old.[40] Hiraoka et al.[46] recently found a significant correlation between myopia progression/axial elongation and many components of corneal HOA. Our results indicate corneal HO RMS to be around 0.30 ± 0.31 µm and 0.28 ± 0.25 µm at baseline respectively in the control and MiSight groups; these values are low, and according to the same study[46], a high change in axial length must be expected. Throughout the 2 years of this study, no differences in either group were found indicating that corneal HOA and SA did not substantially influence the regulation of myopia progression over time; nor were they significantly affected or regulated by dual focus MFCL approach to myopia management.

In conclusion, this study shows no differences between the inherent total and corneal aberrations in children who have been using dual-focus MFCL and single-vision spectacles over 2 years. However, the results show an increment in RMS total in the control group that was due the higher myopia progression and axial elongation seen in this group and reported in previous MASS study. Also the naked eye results in present study indicate that children do not develop relevant corneal aberration changes in response of wearing MiSight contact lens along the time.

## Data Availability

ClinicalTrials.gov Identifier: NCT01917110.
